# Supplementary material for: Causal Relationship between Aging and Anorexia Nervosa: A White-Matter-Microstructure-Mediated Mendelian Randomization Analysis
Source: Biomedicines. 2024 Aug 16;12(8):1874. doi: 10.3390/biomedicines12081874 (PMC11351342; doi:10.3390/biomedicines12081874)
Supplement: Supplementary file 1 [file biomedicines-12-01874-s001.zip › Supplementary Figure/Figure S8/Leaveoneout plot of MR analysis for TL to ubm-b-2063.pdf]

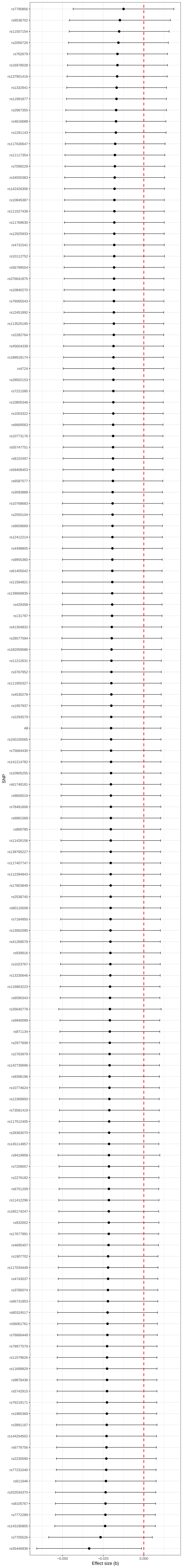

rs7790856
rs6536702
rs11557154
rs2056726
rs762679
rs16978028
rs137901416
rs1332941
rs11991877
rs2967355
rs4616688
rs1291143
rs117630647
rs11117354
rs7099229
rs34550383
rs142426306
rs10845387
rs111527438
rs11769630
rs12925933
rs4731541
rs10112752
rs56799554
rs376641875
rs10840270
rs76065543
rs12451892
rs113525195
rs2282764
rs45604339
rs188918174
rs4724
rs28502153
rs7221585
rs10805346
rs1003322
rs6669563
rs10773176
rs55747751
rs8102497
rs59409453
rs6587577
rs3093888
rs10768683
rs2555104
rs6659669
rs12412214
rs4498805
rs9955360
rs61405042
rs11584821
rs139669835
rs429358
rs131797
rs41304832
rs28577594
rs182059586
rs11212631
rs3767952
rs111950327
rs4530278
rs1957937
rs2293579
All
rs150150565
rs75664430
rs141214782
rs10905255
rs61748181
rs9600019
rs78491606
rs6881568
rs869785
rs11426156
rs139795227
rs117407747
rs112394943
rs17803849
rs2538745
rs80116508
rs7164950
rs13062095
rs41269079
rs939916
rs1023767
rs13230646
rs116863223
rs6590343
rs35640778
rs9940099
rs871134
rs2977608
rs2763979
rs142730696
rs9398196
rs10774624
rs12369950
rs73581419
rs117512405
rs28363070
rs145114957
rs9419958
rs7209057
rs2276182
rs6751209
rs11412296
rs185174247
rs932002
rs17677991
rs4695407
rs1907702
rs117034449
rs4743037
rs3785074
rs66731853
rs80324517
rs56061761
rs76666449
rs79977579
rs11579626
rs11699829
rs9878436
rs5742915
rs76219171
rs1985369
rs3891167
rs144204502
rs6776756
rs2230590
rs77231040
rs611646
rs202034370
rs8105767
rs7772289
rs143190905
rs7705526
rs35446936
SNP
-0.050
-0.025
0.000
0.025
Effect size (b)
